# T266M variants of ANGPTL4 improve lipid metabolism by modifying their binding affinity to acetyl-CoA carboxylase in obstructive sleep apnea

**DOI:** 10.1080/07853890.2024.2337740

**Published:** 2024-04-04

**Authors:** Xinyi Li, Chenyang Li, Wenjun Xue, Zhicheng Wei, Hangdong Shen, Kejia Wu, Huaming Zhu, Huajun Xu, Xiaolin Wu, Hongliang Yi, Jian Guan, Shankai Yin

**Affiliations:** a Department of Otorhinolaryngology Head and Neck Surgery, Shanghai Sixth People’s Hospital Affiliated to Shanghai Jiao Tong University School of Medicine, Shanghai Key Laboratory of Sleep Disordered Breathing, Otorhinolaryngology Institute of Shanghai JiaoTong University, Shanghai, China; b Department of Otorhinolaryngology Head and Neck surgery, Shanghai Eighth People’s Hospital Affiliated to Jiangsu University, Shanghai, China; c Central Laboratory of Shanghai Eighth People’s Hospital, Xuhui Branch of Shanghai Sixth People’s Hospital, P. R. China

**Keywords:** Angiopoietin-like protein 4, obstructive sleep apnea, T266M, ACACA, molecular docking, molecular dynamic simulation

## Abstract

**Background:**

Angiopoietin-like protein 4 (ANGPTL4) is recognized as a crucial regulator in lipid metabolism. Acetyl-CoA carboxylases (ACACAs) play a role in the β-oxidation of fatty acids. Yet, the functions of ANGPTL4 and ACACA in dyslipidemia of obstructive sleep apnea (OSA) remain unclear.

**Methods:**

This study included 125 male OSA subjects from the Shanghai Sleep Health Study (SSHS) who were matched for age, body mass index (BMI), and lipid profile. Serum ANGPTL4 levels were measured *via* ELISA. The ANGPTL4 T266M variants of 4455 subjects along with their anthropometric, fasting biochemical, and standard polysomnographic parameters were collected. Linear regression was used to analyze the associations between quantitative traits and ANGPTL4 T266M. Molecular docking and molecular dynamic simulation were employed to compare the effects of the wild-type ANGPTL4 and its T266M mutation on ACACA.

**Results:**

Serum ANGPTL4 levels significantly decreased with increasing OSA severity (non-OSA: 59.6 ± 17.4 ng/mL, mild OSA: 50.0 ± 17.5 ng/mL, moderate OSA: 46.3 ± 15.5 ng/mL, severe OSA: 19.9 ± 14.3 ng/mL, respectively, *p* = 6.02 × 10^−16^). No associations were found between T266M and clinical characteristics. Molecular docking indicated that mutant ANGTPL4 T266M had stronger binding affinity for the ACACA protein, compared with wild-type ANGPTL4. In terms of protein secondary structure, mutant ANGTPL4 T266M demonstrated greater stability than wild-type ANGPTL4.

**Conclusions:**

Serum ANGTPL4 levels were significantly decreased in OSA patients, particularly among individuals with severe OSA. Although functional ANGTPL4 T266M variants were not associated with lipid levels in OSA, ANGTPL4 T266M could enhance binding affinity for the ACACA protein, potentially regulating lipid metabolism.

## Background

Obstructive sleep apnea (OSA) is an increasingly prevalent sleep disorder characterized by repeated episodes of complete or partial collapse of the upper airway during sleep, leading to fragmented sleep and chronic intermittent hypoxia (CIH) [1, 2] and increased risks of dyslipidemia, atherosclerosis, and cardiovascular diseases (CVDs) [3–5]. In a previous study, we revealed that OSA and dyslipidemia evolved in a complex multistage and nonmonotonic manner [6]. This suggests that the risk for dyslipidemia does not linearly increase with the severity of OSA and that important proteins/molecules regulating lipid metabolism are involved in the severe stage of OSA.

The angiopoietin-like family, particularly angiopoietin-like protein 4 (ANGPTL4) can inhibit lipoprotein lipase (LPL) and has a significant role in lipid metabolism [7, 8]. Elevated serum ANGPTL4 levels were correlated with several metabolic diseases, such as type 2 diabetes mellitus (T2DM), obesity, and CVD [9, 10]. Studies on rodents have shown that CIH upregulates ANGPTL4 levels, thereby inactivating LPL and inhibiting triglyceride (TG) clearance in adipose tissue [11, 12]. Furthermore, expression of the ANGPTL4 gene in endothelial cells is induced by hypoxia [13]; in a small group of OSA subjects in a previous study, serum levels of ANGPTL4 were increased [14]. Thus, we hypothesize that functional polymorphisms in the ANGPTL4 gene are involved in the regulation of dyslipidemia in OSA.

Multiple large-scale studies have linked ANGPTL4 sequence variants to lipoprotein concentrations [15, 16]. Specifically, the rs1044250 (c.797C > T, T266M) variant of ANGPTL4 has been proven to affect TG metabolism [17]. The impact of ANGPTL4 T266M on dyslipidemia has been studied in metabolic diseases such as T2DM [18], but it does not significantly affect the development of prediabetic phenotypes in the white population [19]. There is increasing evidence that ANGPTL4 T266M significantly affects and predicts the risk of CVD [10, 18]. However, the contributions of ANGPTL4 genetic variants to dyslipidemia in OSA are not well understood.

The enzyme acetyl-CoA carboxylase (ACACA, EC 6.4.1.2) catalyzes the carboxylation of acetyl-CoA to malonyl-CoA in lipogenic tissues and is highly expressed in white and brown fat and the liver [20]. Malonyl-CoA is the major precursor for fatty acid synthesis and plays a pivotal role in controlling mitochondrial fatty acid β-oxidation [21]. ANGPTL4 treatment increases the phosphorylation of AMPK and ACACA, as well as the mitochondrial maximum respiratory capacity [22]. Transcriptome analysis and quantitative reverse transcription polymerase chain reaction have revealed that ANGPTL4 and ACACA play a crucial role in response to hypobaric hypoxia [23]. Furthermore, neonatal rat cardiac myocytes under hypoxia showed substantial ACACA activity [24]. In human hepatoma cell lines, hypoxia increases de novo lipogenesis, in parallel with increases in hepatic gene expression of ACACA-1 and fatty acid synthase [25]. Moreover, CIH upregulates multiple genes controlling cholesterol and fatty acid biosynthesis, predominantly fatty acid biosynthesis (ACACA) in obese mice [26]. Nonetheless, complex relationships among CIH, ANGPLT4, and ACACA and the role of functional ANGPLT4 T266M variants in regulating lipid metabolism remain unclear.

Based on the genomic and biochemical data from the Shanghai Sleep Health Study (SSHS), we investigated the role of ANGPLT4 T266M on ACACA in OSA.

## Materials and methods

### Study samples

The present investigation was based on a subset of participants from the SSHS, a study that has been ongoing since 2007 to explore the genetic and environmental risk factors associated with OSA. Details of SSHS were previously published in our genome-wide association study (GWAS) [27]. Participants who underwent laboratory-based polysomnography (PSG), anthropometric and biochemical measurements, and a series of sleep-related questionnaires were included. The exclusion criteria were as follows: history of continuous positive airway pressure (CPAP) treatment or upper airway surgery; nocturnal oxygen or oral appliance usage; use of lipid-lowering drugs; missing data; and presence of other sleep disorders.

To mitigate potential negative feedback effects of lipids on ANGPTL4, 6,433 participants were drawn from a subset of the SSHS cohort and stringently matched for age, body mass index (BMI), high-density lipoprotein cholesterol (HDL-C), low-density lipoprotein cholesterol (LDL-C), TG, total cholesterol (TC), apolipoprotein A (APOA), and apolipoprotein B (APOB). We ultimately included 33 non-OSA, 31 mild, 31 moderate, and 30 severe male OSA participants to evaluate serum concentrations of ANGPTL4. The aforementioned confounding factors were comparable among these groups. The patient selection flow chart is presented in Supplementary Figure S1.

To investigate the genetic impact of ANGPTL4 on CIH-induced dyslipidemia, the most widely studied ANGPTL4 variants (T266M, E40K) of 5,433 participants were extracted from our genomic database between 2007 and 2016. To construct the genomic database for our GWAS, we enrolled newly diagnosed patients from our sleep center who were aged > 18 years; had undergone whole-night standard polysomnography; had not previously received OSA treatment; and had completed comprehensive questionnaires evaluating subjective sleep quality, demographic variables, health status, family disease history, medical history, and lifestyle factors. We excluded patients with missing AHI, ODI, or MAI, other sleep disorders (such as restless leg syndrome and narcolepsy), systemic diseases (e.g. chronic pulmonary, renal, or hepatic failure), cancer, psychiatric disease, hyperparathyroidism, hypoparathyroidism, or polycystic ovarian syndrome. In this study, Female participants, subjects who were missing for T266M, BMI and WHR data were also excluded. Finally, 4,455 patients were included in the study. The patient selection flow chart is presented in Supplementary Figure S2. Besides, only ANGPTL4 variant T266M was analyzed, as the call rates were > 95%, minor allele frequencies > 1%, and they met linkage disequilibrium (LD) criteria of < 0.2.

Written informed consent was obtained from all participants, and the study protocol was approved by the Internal Review Board of the Institutional Ethics Committee of Shanghai Jiao Tong University Affiliated Sixth People’s Hospital. The study protocol was registered with the Chinese Clinical Trial Registry (ChiCTR1900025714).

### Polysomnographic evaluation and OSA definition

A laboratory-based polysomnographic device (Alice 4 or 5; Respironics, Pittsburgh, PA, USA) was used to monitor respiratory events according to the 2012 criteria of the American Academy of Sleep Medicine (AASM) [28]. Sleep was monitored by electroencephalograms (EEG), left and right electrooculograms (EOG), submental electromyograms (EMG), nasal and oral airflow, snoring, electrocardiograms (ECG), thoracic/abdominal movement, pulse oxygen saturation, and body position. Apnea was defined as a 90% reduction in airflow from baseline that lasted ≥ 10 s, while hypopnea was defined as ≥ 30% reduction in airflow in conjunction with either *a* ≥ 3% decrease in oxyhemoglobin saturation or an arousal. The Apnea-Hypopnea Index (AHI) was calculated by the number of apnea and hypopnea events per hour during sleep and was the primary measure to define the severity of OSA. Cut-off AHI levels of 5, 15, and 30 were used to stratify mild, moderate, and severe OSA, respectively.

### Anthropometric and biochemical measurements

Waist circumference (WC), neck circumference (NC), and hip circumference (HC) were measured as previously described [6]. BMI was calculated as weight in kilograms divided by height in meters squared (kg/m^2^). Systolic and diastolic blood pressure (SBP and DBP) were measured in triplicate on the same day after a rest period of at least 10 min, using an automated electronic device (Omron Model HEM-752 Fuzzy, Omron Company). For each participant, a fasting blood sample was drawn from the antecubital vein the morning after polysomnographic monitoring. Fasting blood glucose (FBG), TC, TG, HDL-C, LDL-C, APOA, APOB, and apolipoprotein E (APOE) were measured in the hospital laboratory using an autoanalyzer (H-7600; Hitachi, Tokyo, Japan). Fasting levels of insulin in the serum were measured using an immunoassay diagnostic system. Insulin resistance was calculated using the homeostasis model assessment method (HOMA-IR) as previously described: fasting serum levels of insulin (μU/mL) × fasting plasma levels of glucose (mmol/L)/22.5.

### Serum ANGPTL levels

Serum ANGPTL4 levels were determined using commercial ANGPTL4 enzyme-linked immunosorbent assay (ELISA) kits (BioVendor, Asheville, NC, USA, Catalogue No: RD19107320R). According to manufacturer data, the assay exhibits a sensitivity of 0.173 ng/mL for human ANGPTL4, with an intra-assay coefficient of variation (CV%) < 5% and inter-assay CV% < 7%.

### SNP genotyping

Three genome-wide genotyping platforms were utilized: Affymetrix Genome-Wide Human SNP Array 6.0 (SNP6.0), Affymetrix Axiom Genome-Wide CHB1 Array Plate and Illumina 1 M Array. The genotyping procedures, quality control measures, and genotype imputation of Chinese data were described in our GWAS [27]. Among variants within the ANGPTL4 loci, T266M, which met the quality control criteria for index SNPs, was selected for further investigation.

### Pathway and protein–protein interaction analysis

A Kyoto Encyclopedia of Genes and Genomes (KEGG) analysis was conducted to explore the roles of ANGPTL4 and ACACA in metabolic pathways. The protein–protein interaction network of ANGPTL4 and ACACA was established using the Search Tool for Retrieval of Interacting Genes/Proteins (STRING) database and the Cytoscape Network Analyzer plugin, setting a low confidence threshold for combined-score in the STRING database.

### Molecular docking

Molecular docking of human ACACA (PDB ID: 4ASI) and ANGTPL4 (PDB ID: 6EUB) was performed. The amino acid sequences of ACACA, ANGTPL4, mutant ANGTPL4 T266M were 1618D-I2374, 184 L-T(266)-M400 and 184 L-M(266)-M400, respectively. Protein-to-protein docking was conducted using the HDOCK program that applies a rigid docking method. HDOCK is an FFT-based global docking program, and its scoring method utilizes a modified shape-based pairwise scoring function, which is an efficient iterative technique. The HDOCK program explores all possible rotations and translations of the ligand proteins to determine the structure with the most complementary shapes. An angular spacing of 15° is employed for rotation sampling, and a grid spacing of 1.2 Å is used for translation search based on FFT.

### Molecular dynamic simulation

Both wild-type ANGTPL4 and mutant ANGTPL4 T266M systems were modelled using all-atom explicit solvent models in the GROMACS-2022 software. To ensure comparability, both systems utilized identical parameter selection methods, which included a temperature of 310 K and a run time of 200 ns. The force field was set as AMBER99SB, the water model as TIP3P, and the C and N termini were assigned as COO- and NH3+, respectively. A minimum distance of 1 nm was maintained between the protein and the system box. The system box was cubic, with dimensions 8.297417*822876*77480 nm³. The total atom counts for the ANGTPL4 and mutant ANGTPL4 T266M systems were 43197 and 43200, respectively, inclusive of two chloride ions. Periodic boundary conditions were applied throughout all simulations.

Prior to MD simulation, the system’s energy was first minimized. Spatial conflicts within the system were resolved using a 50,000 step steepest descent method. Subsequently, simulations were conducted under NVT and NPT (1 bar) integrations, each running for 0.1 ns to balance the system. Pressure parameters were set using the Parrinello-Rahman method to maintain a pressure of 1 bar. Temperature parameters were coupled to an external heat bath with a velocity rescale coupling method to maintain a steady temperature of 310 K. The neighbour list was updated every 10 steps, with a 1.0 nm cutoff distance using the Verlet buffer. All bond lengths were restrained using the LINCS method (for proteins) and the SETTLE algorithm (for water molecules), with an integration time of 2 fs. The cutoff value for van der Waals interaction was set at 1.0 nm. The electrostatic interaction was computed using the particle mesh Ewald (PME) method, with a real space cutoff value set at 1.0 nm.

Main chain root mean square deviation (RMSD), residue-based root mean square fluctuation (RMSF), radius of gyration (Rg), solvent accessible surface area (SASA), and hydrogen bond (H-bond) number were employed to characterize the structural and conformational properties of both wild-type ANGPTL4 and the mutant ANGPTL4 T266M.

Secondary structures of proteins were calculated using the Define Secondary Structure of Proteins (DSSP) software, which categorized structures into coil, β-sheet, β-bridge, bend, turn, α-helix, 5-helix, and 3-helix. A hydrogen bond was considered to form when the distance between the donor D and acceptor A was less than or equal to 0.35 nm, and the included angle between donor D-H and acceptor A was less than or equal to 150°.

### Statistical analysis

Data that were normally distributed are presented as means ± standard deviations (SDs), while categorical data were presented as percentages. Differences in baseline characteristics among groups were analyzed using the least-significant difference (LSD) test, one-way analysis of variance (ANOVA), or the χ2 test, based on the data distribution characteristics. We employed MATLAB software (Version 8.0, MathWorks, USA) for the matching method, thereby enhancing comparability between groups. Nonparametric tests were used to compare clinical characteristics in recessive models of different OSA severities. Associations between quantitative traits and ANGPTL4 T266M were examined through linear regression, and standardized regression coefficients were presented. Unless otherwise specified, statistical analyses were carried out using SPSS v.21.0.0 (SPSS, Chicago, Illinois, USA). Two-tailed p values < 0.05 were considered to indicate statistical significance.

## Results

### Serum ANGPTL4 levels decreased with OSA severity

After matching for age, BMI, TC, TG, HDL-C, LDL-C, APOA, and APOB, 125 male subjects (33 non-OSA, 31 mild OSA, 31 moderate OSA, 30 severe OSA) were included in the study. The participants’ basic characteristics are presented in Table 1. Fasting blood glucose, fasting blood insulin, and HOMA-IR showed increasing trends with OSA severity (*p* < 0.05). The serum level of ANGPTL4 significantly decreased with increasing OSA severity (non-OSA: 59.6 ± 17.4 ng/mL, mild OSA: 50.0 ± 17.5 ng/mL, moderate OSA: 46.3 ± 15.5 ng/mL, severe OSA: 19.9 ± 14.3 ng/mL, respectively, *p* = 6.02 × 10^-16). Based on these data, we concluded that ANGPTL4 levels were predominantly affected by AHI and minimum SaO_2_, particularly in the severe OSA group, suggesting that CIH might affect ANGPTL4 levels. Spearman’s test was used to analyze correlations of serum ANGPTL4 levels with obesity, lipid levels, and sleep-related breathing parameters. ANGPTL4 levels were negatively associated with NC, AHI, and ODI (r= −0.195, *p* = 0.031; r= −0.599, *p* < 0.001; r= −0.488, *p* < 0.001, respectively); they were positively associated with minimum SaO_2_ (r= −0.390, *p* < 0.001) (Supplementary Table S1).

**Table 1.** Baseline characteristics of 125 matched participants with different OSA severity levels.

	Non-OSA*	Mild OSA*	Moderate OSA*	Severe OSA*	P
Number	33	31	31	30	–
Age (years)	41.0 ± 8.1	39.3 ± 7.5	40.0 ± 8.5	40.1 ± 8.1	0.846
Body mass index (Kg/m^2^)	25.1 ± 1.9	25.4 ± 2.2	25.4 ± 2.2	25.3 ± 1.8	0.957
SBP (mm/Hg)	118.2 ± 17.9	115.6 ± 11.0	123.2 ± 18.6	115.5 ± 13.4	0.192
DBP (mm/Hg)	78.7 ± 11.9	74.6 ± 8.1	78.6 ± 12.1	75.9 ± 9.0	0.338
Neck circumference (cm)	38.5 ± 2.4	39.0 ± 2.4	39.0 ± 2.2	39.6 ± 2.1	0.299
Waist circumference (cm)	91.8 ± 7.3	90.2 ± 6.4	92.7 ± 5.7	94.0 ± 5.1	0.120
Hip circumference (cm)	98.8 ± 5.4	97.2 ± 5.3	98.5 ± 4.3	98.6 ± 4.1	0.606
Wasit/hip circumference ratio	0.93 ± 0.05	0.93 ± 0.04	0.94 ± 0.04	0.95 ± 0.04	0.061
TC (mg/dl)	179.2 ± 27.3	187.8 ± 36.5	193.3 ± 40.4	179.8 ± 27.5	0.283
TG (mg/dl)	146.0 ± 85.1	146.5 ± 87.3	159.8 ± 73.2	133.9 ± 49.8	0.618
HDL-C (mg/ml)	39.9 ± 8.0	41.3 ± 6.9	40.1 ± 6.7	40.6 ± 8.4	0.868
LDL-C (mg/dl)	111.0 ± 23.7	121.1 ± 32.7	119.3 ± 34.4	112.1 ± 24.4	0.417
APOA(g/l)	1.05 ± 0.20	1.06 ± 0.15	1.03 ± 0.15	1.01 ± 0.15	0.640
APOB (g/l)	0.80 ± 0.11	0.87 ± 0.14	0.88 ± 0.18	0.83 ± 0.12	0.092
APOE (mg/dl)	3.97 ± 1.31	4.07 ± 1.40	4.52 ± 1.72	3.98 ± 0.85	0.331
Fasting blood glucose (mmol/l)	4.7 ± 0.9	5.3 ± 0.5	5.3 ± 1.0	5.2 ± 0.6	**0.011**
Fasting blood insulin (uU/ml)	8.9 ± 3.7	8.3 ± 3.9	12.0 ± 6.0	9.4 ± 4.1	**0.009**
HOMA-IR	1.88 ± 0.93	1.99 ± 1.05	2.85 ± 1.56	2.22 ± 1.06	**0.007**
Apnea-hypopnea index	1.8 ± 1.2	9.9 ± 2.7	21.7 ± 4.6	55.1 ± 13.5	**2.44 × 10^-58^**
Oxygen desaturation index	2.4 ± 2.6	9.0 ± 5.0	24.0 ± 12.8	43.4 ± 17.8	**3.50 × 10^-30^**
Minimum SaO2	92.4 ± 3.6	85.3 ± 6.8	81.2 ± 9.4	70.7 ± 11.1	**2.07 × 10^-17^**
ANGPT4L (ng/ml)	59.6 ± 17.4	50.0 ± 17.5	46.3 ± 15.5	19.9 ± 14.3	**6.02 × 10^-16^**

Data are presented as mean values ± SD.

TG: triglycerides; TC: total cholesterol; HDL-C: high-density lipoprotein cholesterol; LDL-C: low-density lipoprotein cholesterol; HOMA-IR: homeostasis model assessment of insulin resistance; APOA: apolipoprotein A; APOB: apolipoprotein B; APOE: apolipoprotein E; SBP: systolic blood pressure; DBP: diastolic blood pressure; SaO2: oxygen saturation.

p values < 0.05 were shown in bold.

*The subgroups of OSA were matched for age, BMI, HDL-C, LDL-C, TC, TG, APOA, APOB, APOE.

### Clinical characteristics of T266M recessive models in OSA subjects

To further investigate the impact of ANGPTL4 genetic variants on lipid profiles in 4455 OSA subjects (678 non-OSA, 894 moderate OSA, and 2883 severe OSA), we analyzed the clinical (anthropometric, biochemical phenotypes, and sleep parameters) characteristics of ANGPTL4 T266M recessive models across different OSA severities using a nonparametric test (Supplementary Table S2). We found that serum levels of HDL-C were higher in the CT + TT group than in the CC group in non-OSA (1.07 [0.96–1.26] mg/mL vs. 1.02 [0.92–1.17] mg/mL, *p* = 0.029). In severe OSA, the CT + TT group had lower HDL-C levels than the CC group (0.96 [0.85–1.07] mg/mL vs. 0.99 [0.87–1.13] mg/mL, *p* = 0.003). The minimum SaO2 was lower in the CT + TT group than the CC group in moderate OSA (82% [75–86%] vs. 83% [78–87%], *p* = 0.01).

We analyzed quantitative traits using linear regression under an additive genetic model. No associations were found between T266M and clinical characteristics, except for HDL-C (β=-0.056, *p* = 0.003) in severe OSA (Table 2).

**Table 2.** Associations between T266M and clinical characteristics of OSA participants.

	Non-OSA		Moderate OSA		severe OSA		Total	
	ß	P	ß	P	ß	P	ß	P
**Demographics**								
age	0.02	0.605	−0.023	0.494	−0.013	0.473	−0.014	0.359
BMI	−0.056	0.148	0.036	0.290	−0.005	0.807	−0.014	0.352
SBP (mm/Hg)	−0.01	0.981	0.042	0.229	−0.005	0.800	0.001	0.966
DBP (mm/Hg)	0.031	0.432	0.046	0.185	−0.014	0.473	−0.002	0.916
NC (cm)	−0.06	0.121	−0.03	0.387	−0.01	0.609	−0.028	0.064
WC (cm)	−0.068	0.081	0.008	0.816	0.023	0.756	−0.005	0.756
HC (cm)	−0.058	0.137	0.012	0.718	0.006	0.756	−0.01	0.528
WHR	−0.046	0.242	−0.003	0.929	0.03	0.120	0.00019	0.990
**Biochemistry assays**								
TC (mg/dl)	0.007	0.865	0.017	0.610	0.006	0.761	0.004	0.814
TG (mg/dl)	−0.014	0.717	0.006	0.859	0.005	0.785	−0.001	0.972
HDL-C (mg/ml)	0.072	0.063	0.031	0.358	−0.056	**0.003**	−0.011	0.474
LDL-C (mg/dl)	0.002	0.963	0.014	0.685	0.007	0.696	0.004	0.800
APOA (g/l)	0.039	0.317	−0.039	0.259	−0.016	0.414	−0.010	0.529
APOB (g/l)	0.025	0.514	0.022	0.521	0.002	0.928	0.004	0.811
APOE (mg/dl)	−0.049	0.205	0.009	0.804	0.008	0.665	−0.003	0.858
FBG (mmol/l)	−0.077	0.048	−0.014	0.690	−0.015	0.440	−0.027	0.074
FIN (uU/ml)	−0.008	0.835	−0.049	0.150	0.001	0.973	−0.016	0.286
HOMA-IR	−0.038	0.328	−0.049	0.155	−0.002	0.928	−0.021	0.966
**Sleep apnea**								
AHI	0.019	0.619	0.008	0.810	0.028	0.139	−0.11	0.467
ODI	−0.048	0.219	0.03	0.369	0.026	0.174	−0.01	0.53
Minimum SaO2	0.002	0.960	−0.05	0.140	0.023	0.222	0.02	0.184
MAI	−0.028	0.463	0.013	0.692	0.025	0.190	0.007	0.628

Data are presented as median and interquartile range (25%-75%).

TG: triglycerides; TC: total cholesterol; HDL-C: high-density lipoprotein cholesterol; LDL-C: low-density lipoprotein cholesterol; HOMA-IR: homeostasis model assessment of insulin resistance; APOA: apolipoprotein A; APOB: apolipoprotein B; APOE: apolipoprotein E; SBP: systolic blood pressure; DBP: diastolic blood pressure; SaO2: oxygen saturation.

p values < 0.05 were shown in bold.

### Protein–protein interactions (PPIs) reveal that ANGPTL4 interacts with ACACA

KEGG metabolic pathway analysis revealed that ANGPTL4 and ACACA are involved in lipid metabolism, including cholesterol metabolism (Supplementary Figure S3) and the fatty acid biosynthesis pathway (Supplementary Figure S4). PPIs, retrieved by the STRING database, suggested that ANGPTL4 interacts with ACACA (Supplementary Figure S5).

### Molecular docking

The rigid docking method was utilized to globally search for binding conformation. The structure with the lowest score was selected as the initial structure for the two complex systems. The binding energy of wild-type ANGPTL4 and ACACA protein was −268.86 kcal/mol, whereas that of mutant ANGPTL4 T266M and ACACA protein was −272.82 kcal/mol. These results indicate that the mutant ANGPTL4 T266M has stronger binding affinity for the ACACA protein, compared with wild-type ANGPTL4.

**Figure 1.**
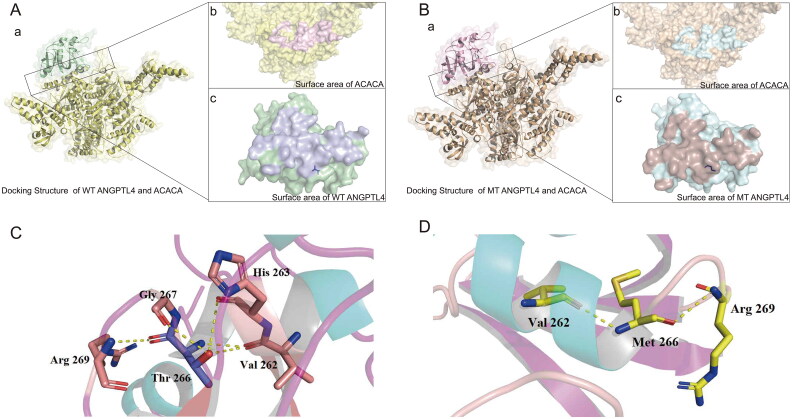
Visualization of the docked complex between human ANGPTL4 and ACACA. A: a) Interaction docking structure between wild-type ANGPTL4 and ACACA. b) Surface area visualization showing the compact fitting of ACACA. c) Surface area visualization showing the compact fitting of wild-type ANGPTL4. B: a) Interaction docking structure between mutant ANGPTL4 T266M and ACACA. b) Surface area visualization showing the compact fitting of ACACA. c) Surface area visualization showing the compact fitting of mutant ANGPTL4 T266M. C: Intra-chain interaction of wild-type ANGPTL4. D: Intra-chain interaction of mutant ANGPTL4 T266M.

The docking structure complexes of ANGPTL4, ANGPTL4 T266M, and ACACA are shown in Figure 1A, B. The interaction residues on the contact surface were analyzed (Table 3). For the interface structure between wild-type ANGPTL4 and ACACA, there were 35 interaction residues on the wild-type ANGPTL4 protein and 35 on the ACACA protein, with a contact area between them of 1305.8 Å^2.^ In the structure between mutant ANGPTL4 T266M and ACACA, there was one less interaction residue on the ACACA protein than on the wild-type ANGPTL4 complex, and the contact area for this structure was also slightly lower (1287.1 Å^2^). The interactions of residue pairs between proteins in the two complexes was analyzed (Supplementary Table S2).

**Table 3.** Interactions between residues located on the contact surfaces of ANGPTL4, T266M, and ACACA.

Protein systems	Wild Type ANGPTL4	ACACA	Mutant ANGTPL4 T266M	ACACA
Residues Number	Gln 189	Asp 1619	Gln 189	Asp 1619
	Phe 192	Leu 1621	Phe 192	Leu 1621
	Gln 193	Gln 1622	Gln 193	Gln 1622
	Arg 197	Arg 1625	**Val 194**	Arg 1625
	Ser 220	Phe 1626	Arg 197	Phe 1626
	Asp 221	Gln 1629	Ser 220	Gln 1629
	Gly 222	Ser 1630	Asp 221	Ser 1630
	Gly 223	Gly 1632	Gly 222	Gly 1632
	Asn 270	Thr 1633	Gly 223	Thr 1633
	Ser 271	Thr 1634	Asn 270	Thr 1634
	Arg 272	Asp 1638	Ser 271	Asp 1638
	Glu 285	Met 1642	Arg 272	Met 1642
	Leu 286	**Gln 1645**	Glu 285	Ile 1648
	Leu 287	Ile 1648	Leu 286	Lys 1649
	Gln 288	Lys 1649	Leu 287	Glu 1652
	Phe 289	Glu 1652	Gln 288	Ser 1653
	Ser 290	Ser 1653	Phe 289	Thr 1656
	Val 291	**Met 1654**	Ser 290	Gln 1657
	His 292	Thr 1656	Val 291	**Glu 1880**
	Thr 305	Gln 1657	His 292	Cys 1924
	Pro 307	Cys 1924	Thr 305	Asp 1925
	Val 308	Asp 1925	Pro 307	Asp 1926
	Gly 310	Asp 1926	Val 308	Phe 1927
	Gln 311	Phe 1927	Gly 310	Glu 1928
	Leu 312	Glu 1928	Gln 311	Phe 1931
	Gly 313	Phe 1931	Leu 312	Thr 1932
	Ala 314	Thr 1932	Gly 313	Arg 1973
	Thr 315	Arg 1973	Ala 314	Ala 1977
	Thr 316	Ala 1977	Thr 315	Trp 1988
	Pro 319	Trp 1988	Thr 316	Tyr 1995
	**Trp 380**	Tyr 1995	Pro 319	Gly 1996
	Thr 382	Gly 1996	Thr 382	Ser 1997
	Trp 383	Ser 1997	Trp 383	Glu 2000
	Gln 398	Glu 2000	Gln 398	Gln 2003
	Met 400	Gln 2003	Met 400	–
Contact area	1305.8 Å^2^		1287.1 Å^2^	

*The residues difference between the two complexes is highlighted in bold.

From the observation of the structure of wild-type ANGPTL4 and mutant ANGPTL4 T266M, it is clear that for the wild-type ANGPTL4, residue Thr 266 can generate five hydrogen bonds with four surrounding residues (Val 262, His 263, Gly 267, Arg 269) (Figure 1C). At residue Met 266 in the mutant ANGPTL4 T266M, hydrogen bonding with residues Val 262, His 263, and Gly 267 disappeared due to the absence of the carbon-oxygen double bond of the side chain, leaving only two hydrogen bonds on the main chain (Val 262, Arg 269) (Figure 1D). This suggests that the presence of residue Thr 266 strengthens the intra-chain interaction of wild-type ANGPTL4.

### Molecular dynamic simulation

**Figure 2.**
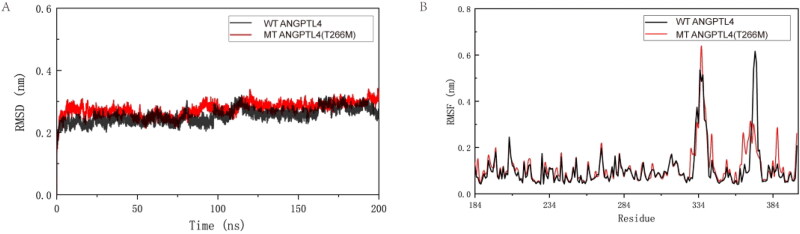
Interpretation of the stability of wild-type (WT) and mutated ANGPTL4. A: Comparison of root mean square deviation (RMSD) for WT and mutated ANGPTL4 proteins. B: Comparison of root mean square fluctuation (RMSF) for residues of WT and mutated ANGPTL4.

A trajectory analysis of the two proteins was carried out to further explore the differences between ANGPTL4 and ANGPT4 T266M proteins using molecular dynamics simulation. Both systems were analyzed at 310 K for 200 ns. Given that residues Thr and Met are both uncharged amino acids, the parameters of the two systems are identical, barring the number of protein atoms. RMSD analysis was conducted on the two protein systems, which converged after 120 ns (Figure 2A). The results (Figure 2B) indicated that the RMSF values for most of the mutant ANGPTL4 T266M protein residues were marginally higher than those of the wild-type ANGPTL4 protein residues. This suggests that most residues in the mutant ANGPTL4 T266M protein exhibit more flexibility. However, the RMSF values of the wild-type ANGPTL4 protein at residues 305–321 and 369–375 were higher than those of the mutant ANGPTL4 T266M, indicating that residues 305–321 and 369–375 in the wild-type ANGPTL4 protein display flexible properties.

**Figure 3.**
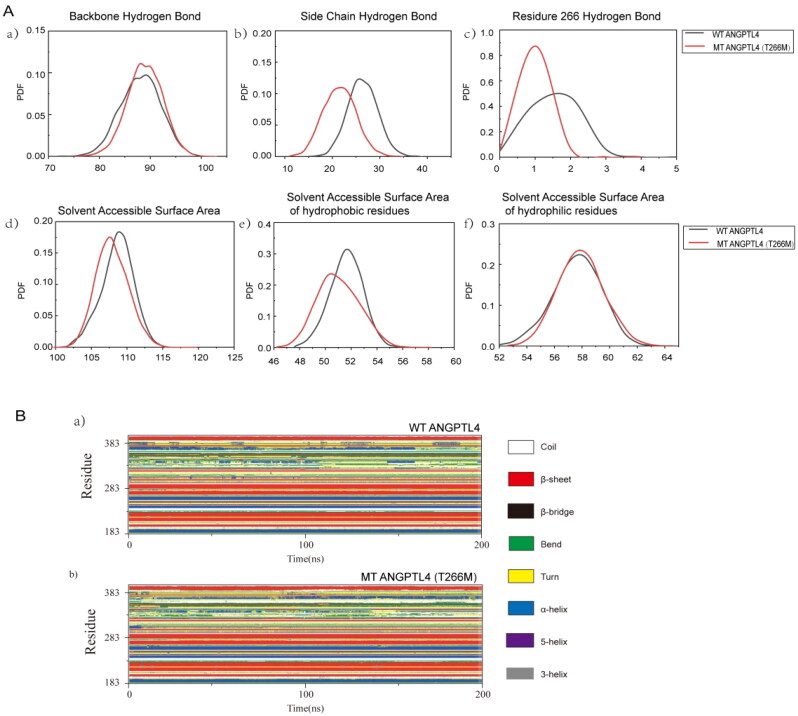
Comparison of the structures of WT and mutated ANGPTL4. A: Probability density function (PDF) map comparing WT and mutated ANGPTL4 in a) backbone hydrogen bonds; b) side chain hydrogen bonds; c) hydrogen bonds at residue 266; d) solvent-accessible surface area; e) solvent-accessible surface area of hydrophobic residues; f) solvent-accessible surface area of hydrophilic residues. B: a) Secondary structure of WT ANGPTL4 protein; b) Secondary structure of mutant (MT) ANGPTL4 protein.

After 120 ns, the number of main chain hydrogen bonds, side chain hydrogen bonds, and hydrogen bonds at residue 266 of the two protein systems were analyzed. From the probability density function diagram of hydrogen bonds, it is evident that the main chain of the mutant ANGPTL4 T266M has one more hydrogen bond than the wild-type ANGPTL4 protein, resulting in a difference of one main chain hydrogen bond between the wild-type ANGPTL4 protein and the mutant ANGPTL4 T266M protein (Figure 3A-a). The peak value of the side chain hydrogen bond for the wild-type ANGPTL4 protein was 27, and that for the mutant ANGPTL4 T266M protein was 23 (Figure 3A-b). Consequently, in these two protein systems, wild-type ANGPTL4 has more hydrogen bonds on the protein side chain than the mutant ANGPTL4 T266M, and the mutant has more hydrogen bonds on the main chain than wild-type ANGPTL4. This suggests that the interaction between wild-type protein residues is reflected in the hydrogen bonds on the side chain. The hydrogen bond at residue 266 was consistent with the initial structure, and the number of hydrogen bonds at residue Thr 266 remained higher than that at residue Met 266 during the molecular dynamic simulation process (Figure 3A-c).

Next, the solvent-accessible surface areas of the wild-type ANGPTL4 and the mutant ANGPTL4 T266M proteins was analyzed. Figure 3A-d displays the probability density function diagram (PDF) of solvent-accessible surface areas for both protein systems. The wild-type ANGPTL4 protein had a greater peak solvent-accessible surface area than the mutant ANGPTL4 T266M protein, indicating that the wild-type ANGPTL4 protein has a more relaxed structure. There are numerical differences in the solvent-accessible surface areas of hydrophobic residues between the two systems, while the solvent-accessible surface area functions of hydrophilic residues are almost identical (Figure 3A-e, f). The greater solvent-accessible surface area of the wild-type ANGPTL4 protein compared with the mutant ANGPTL4 T266M suggests that the structure of the wild-type ANGPTL4 protein is more relaxed, allowing hydrophobic residues to be exposed and come into contact with solvents.

The secondary structure of both systems remained relatively stable before residue 283, with less structural transformation occurring. However, after that residue, changes in the secondary structure became more pronounced (Figure 3B). At residues 303–313, the helical structure of both systems transitioned from an α-helix to a 3-helix. The residues at 323–328 displayed a β-bridge structure in the wild-type ANGPTL4 protein system, whereas in the mutant ANGPTL4 T266M protein, the β-bridge ultimately evolved into a β-sheet structure. At residues 369–376, the wild-type ANGPTL4 protein exhibited a transition from a 3-helix to an α-helix, ultimately changing to a bend and turn, whereas the helix structure of the mutant ANGPTL4 T266M protein transitioned from a 3-helix to an α-helix, with the residues involved in the helix structure gradually increasing. At residue 383, the helix structure of both protein systems was unstable, with no helix structure forming by the end of the simulation. The helix result of the wild-type ANGPTL4 protein in residues 369–376 also elucidates its high RMSF value and increased solvent-accessible surface area of hydrophobic residues. Due to the transition from a helix to a bend and turn, these residues lost their original bond, which makes the residues more flexible and expands the contact area between the internal hydrophobic residues and the solvent. In terms of the types of secondary structures generated, the mutant ANGPTL4 T266M protein was more stable than the wild-type ANGPTL4 protein.

## Discussion

We observed a significant decrease in serum levels of ANGPTL4 due to CIH in a well-matched study. We investigated the role of ANGPTL4 in regulating lipid disorder OSAHS by introducing a functional SNP of ANGPTL4 to explore the impact of ANGPTL4 on blood lipids and its mechanism from a genetic perspective. However, no associations were found between ANGPTL4 T266M and OSA-related parameters. Therefore, we hypothesized that CIH might affect the binding ability of wild-type and mutant ANGPTL4 to downstream proteins, consequently impacting lipid metabolism. Through molecular docking, we discovered that the mutant ANGPTL4 T266M exhibited stronger binding affinity for the ACACA protein compared with the wild-type ANGPTL4. The mutant ANGPTL4 T266M protein maintained a stable overall main chain structure and secondary structure during the molecular dynamic simulation process.

Multiple OSA-related factors, such as hypoxia and obesity, result in changes in serum levels of ANGPTL4. In turn, ANGPTL4 plays critical roles in lipid metabolism [9]. However, the negative feedback of lipid levels on ANGPTL4 levels cannot be overlooked. Feedback regulation of lipids is a physiological process that enables humans and other animals to adapt to changes and maintain their serum levels of lipids within relatively narrow limits [29, 30]. Therefore, to mitigate the effects of these confounders and to reveal the impacts of CIH on ANGPTL4 levels, we strictly matched for age, BMI, and particularly their lipid profile (HDL-C, LDL-C, TC, TG, APOA, and APOB) in different OSA subgroups. Our data suggest that CIH can decrease ANGPTL4 levels. Muniesa et al. reported that hypoxia stimulated the expression and secretion of ANGPTL4 *in vitro* [31]. Other studies have also shown that ANGPTL4 is upregulated at both the protein and mRNA levels under hypoxic conditions in epididymal white adipose tissue [12] and other tissues [13, 32]. This upregulation of ANGPTL4 might be a key factor for hypoxia-derived LPL inactivation and inhibited TG uptake [12, 32, 33]. The effect of ANGPTL4 on dyslipidemia was transient and attenuated at the later stage [34, 35]. This suggests that ANGPTL4 is upregulated in the early stages of CIH, leading to elevated serum levels of TGs and TC, and that the effects of ANGPTL4 were reduced at the later stage during long-term hypoxia. Another possible reason for the decreased ANGPTL4 level in OSA was that CIH may accumulate TG storage in adipocytes by upregulating ANGPTL4 [33, 36], and simultaneously inhibit ANGPTL4 secretion from adipose tissues to blood circulation to reach a new dynamic equilibrium under CIH conditions.

A previous study showed that T266M variants are associated with lower TG levels in the context of T2DM in the Look Action for Health in Diabetes Clinical Trial [37]. Similarly, T266M variants not only reduced fasting TG levels but also indicated a decreased cardiovascular risk in T2DM Tunisian patients (17). However, in the Northwick Park Heart Study, T266M demonstrated no association with plasma TG but was correlated with ANGPTL4 levels [38]. Another study found no significant correlation between T266M and plasma lipid measures, insulin sensitivity, and insulin secretion in nondiabetic subjects [19]. Although the aforementioned studies did not account for the coordinating roles of ANGPTL4 in dyslipidemia and did not consider both ANGPTL4 variants simultaneously, they demonstrated a tendency for T266M variants to associate with TG and other metabolic variables. In our study, we found no associations between T266M and lipid indicators in OSA. Thus, we focused on the binding ability of wild-type and mutant ANGPTL4 to ACACA, which may subsequently regulate lipid metabolism.

Dysregulation of ANGPTL4 in podocytes under hyperlipidemia may be enacted through the AMPK/ACACA signalling pathway [39], which plays a crucial role in exercise-induced AMPK/ACACA activation in skeletal muscle [22]. Both *in vivo* and *in vitro* studies have shown that the role of ANGPTL4 may be activated through the ACACA signaling pathway, consistent with our PPI results. However, fewer studies have focused on the effects of ANGPTL4 functional variants on ACACA. The mutant ANGPTL4 T266M protein was stable and exhibited stronger binding affinity for the ACACA protein, compared with wild-type ANGPTL4, suggesting that T266M variants can play roles in lipid metabolism. The underlying mechanisms require further exploration.

Our study had some limitations that should be considered when interpreting the results. First, we could not establish a causal relationship because causality can only be assessed in interventional settings. Because the subjects came from a clinical center, there might have been a risk of selection bias. Second, the index SNPs of ANGPTL4 may have contributed to variability in lipid metabolism; however, additional functional studies will be required to explore this possibility. Third, the mechanisms underlying the *in vivo* and *in vitro* lipid metabolism of ANGPTL4 and its T266M variants were not examined in depth. Based on the results of this preliminary study, *in vitro* studies are needed to explore the functions of ANGPTL4 and ACACA.

## Conclusion

Serum levels of ANGPTL4 were lower in OSA subjects but ANGPTL4 T266M variants were not associated with lipid indicators. ANGPTL4 T266M had stronger binding affinity for the ACACA protein, compared with wild-type ANGPTL4. These data suggest that the ANGPTL4 T266M variant can alter its binding affinity for ACACA to regulate lipid metabolism.

## Supplementary Material

Supplemental Material

## Data Availability

All data during this study can provide if needed.

## Abbreviations

ANGPTL: Angiopoietin-like protein
OSA: obstructive sleep apnea
CIH: chronic intermittent hypoxia
ACACA: Acetyl-CoA Carboxylase
TG: triglycerides
TC: total cholesterol
HDL-C: high-density lipoprotein cholesterol
LDL-C: low-density lipoprotein cholesterol
CVD: cardiovascular disease
LPL: lipoprotein lipase
T2D: type 2 diabetes mellitus
PDF: probability density function
PSG: polysomnography
APOA: apolipoprotein A
APOB: apolipoprotein B
APOE: apolipoprotein E
ELISA: enzyme-linked immunosorbent assay
IR: insulin resistance
SNP: single nucleotide polymorphism
AHI: apnea-hypopnea index
SaO2: oxygen saturation
RMSD: mean square deviation
RMSF: root mean square fluctuation;
