# Supplementary material for: T266M variants of ANGPTL4 improve lipid metabolism by modifying their binding affinity to acetyl-CoA carboxylase in obstructive sleep apnea
Source: Ann Med. 2024 Apr 4;56(1):2337740. doi: 10.1080/07853890.2024.2337740 (PMC10997356; doi:10.1080/07853890.2024.2337740)
Supplement: Supplemental Material [file IANN_A_2337740_SM3493.zip › Supplementary_Figure_S3 (1).pdf]

CHOLESTEROL METABOLISM

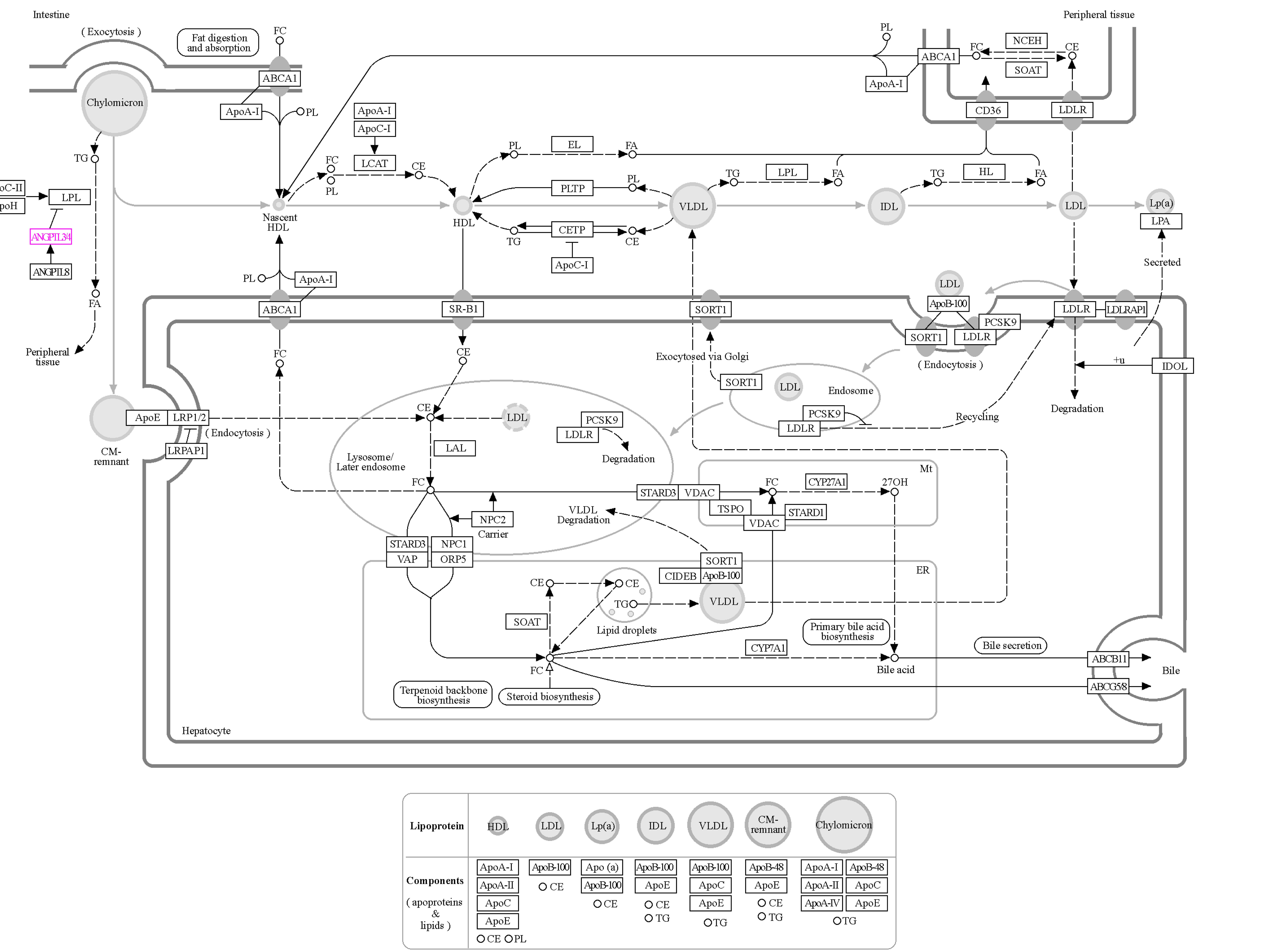

(c) Kanehisa Laboratories
