# Supplementary material for: T266M variants of ANGPTL4 improve lipid metabolism by modifying their binding affinity to acetyl-CoA carboxylase in obstructive sleep apnea
Source: Ann Med. 2024 Apr 4;56(1):2337740. doi: 10.1080/07853890.2024.2337740 (PMC10997356; doi:10.1080/07853890.2024.2337740)
Supplement: Supplemental Material [file IANN_A_2337740_SM3493.zip › Supplementary_material (1).pdf]

Supplementary Table S1. Relationships of serum ANGPTL4 levels with obesity, lipid levels, and sleep-related breathing parameters.

	Non-OSA			Mild OSA			Moderate OSA			Severe OSA			Total	
	r	P		r	P		r	P		r	P		r	P
**Demographics**														
BMI	-0.540	0.765		-0.053	0.775		-0.053	0.779		-0.102	0.591		-0.067	0.460
SBP (mm/Hg)	-0.346	0.052		-0.158	0.405		-0.106	0.577		0.302	0.111		-0.090	0 328
DBP (mm/Hg)	-0.311	0.084		-0.070	0.711		0.009	0.962		0.234	0.222		-0.049	0 595
NC (cm)	-0.043	0.813		0.048	0.805		-0.097	0.602		-0.104	0.583		-0.195	**0.031**
WC (cm)	-0.064	0.724		-0.075	0.698		-0.049	0.794		-0.118	0 534		-0.153	0.091
HC (cm)	-0.171	0.341		-0.001	0.996		-0.071	0.703		-0.033	0.863		-0.042	0.648
WHR	0.045	0.803		0.062	0.749		-0.114	0.540		-0.193	0.307		0.171	0.058
**Biochemistry assays**														
TC (mg/dl)	0.204	0.255		0.133	0.475		0.166	0.371		-0.019	0.921		0.134	0.137
TG (mg/dl)	-0.417	**0.016**		0.263	0.153		-0.185	0.319		-0.063	0.741		-0.080	0.375
HDL-C (mg/ml)	0.302	0.087		-0.179	0.337		0.334	0.667		0.012	0.949		0.064	0 480
LDL-C (mg/dl)	0.148	0.410		0.134	0.473		0.279	0.128		0.043	0.822		0.115	0.202
APOA(g/l)	0.274	0.123		0.096	0.607		-0.130	0.486		0.058	0.762		0.110	0.222
APOB (g/l)	-0.020	0.912		0.246	0.183		0 195	0.294		0.040	0.833		0.086	0.339
APOE (mg/dl)	-0.194	0.279		0.417	**0.020**		-0.097	0.605		0.129	0.498		0.039	0.664
FBG (mmol/l)	-0.368	**0.035**		0.228	0.218		0.268	0 145		0.397	**0.030**		-0.088	0.332
FIN (uU/ml)	-0.059	0.745		0.088	0.640		0.093	0.618		0.173	0.360		-0.018	0.834
HOMA-IR	-0.249	0.162		0.068	0.717		0.278	0130		0.193	0307		-0 057	0.531
**Sleep apnea**														

AHI	0.113	0.531		0.152	0.414		-0.132	0 478		-0.310	0.095		-0.599	**< 0.001**
ODI	0.083	0.648		0.081	0.665		0.024	0.905		-0.227	0.237		-0.488	**< 0.001**
Minimum SaO2	-0.154	0.391		-0.430	**0.016**		0.054	0.787		-0.044	0.819		0.390	**< 0.001**

Supplementary Table S2 Clinical characteristics of the different severity OSA and non-OSA population (T266M)

	Non-OSA (N=678)			Moderate OSA (N=894)			Severe OSA (N=2883)		
	CC (n=586)	CT+TT (n=92)	P	CC (n=786)	CT+TT (n=108)	P	CC (n=2570)	CT+TT (n=313)	P
**Demographics**									
Age (years)	35(29-45)	34(30-45)	0.711	42(34-53)	41(34-50)	0.435	42(35-51)	41(33-52)	0.332
BMI (Kg/m2)	24.22(22.38-26.17)	23.75(21.84-25.54)	0.138	26.14(24.43-28.38)	26.45(24.69-28.97)	0.330	27.76(25.83-30.07)	27.76(25.39-30.30)	0.963
SBP (mm/Hg)	120(113-129)	120(112-130)	0.884	124(116-135)	130(116-138)	0.134	128(120-138)	126(120-136)	0.737
DBP (mm/Hg)	78(71-82)	80(73-84)	0.171	80(72-86)	80(73-90)	0.292	81(76-90)	80(76-90)	0.516
NC (cm)	38(36-40)	38(36-40)	0.196	40(38-42)	4038-42()	0.530	41(39-43)	41(39-43)	0.905
WC (cm)	90(84-95)	88(83-95)	0.210	95(91-101)	96(92-100)	0.754	100(94-106)	101(95-108)	0.07
HC (cm)	98(94-102)	97(93-100)	0.171	100(97-105)	101(97-106)	0.669	103(99-107)	104(99-108)	0.351
WHR	0.91(0.88-0.95)	0.92(0.86-0.95)	0.647	0.95(0.92-0.98)	0.95(0.92-0.98)	0.959	0.97(0.94-1.00)	0.97(0.94-1.01)	0.059
**Biochemistry assays**									
TC (mg/dl)	4.37(3.81-4.99)	4.38(3.88-4.91)	0.847	4.67(4.11-5.28)	4.65(4.18-5.38)	0.718	4.81(4.25-5.43)	4.78(4.19-5.48)	0.843
TG (mg/dl)	1.29(0.83-1.90)	1.18(0.88-1.77)	0.311	1.63(1.18-2.33)	1.65(1.11-1..14)	0.740	1.82(1.30-2.64)	1.71(1.24-2.60)	0.252
HDL-C (mg/ml)	1.03(0.91-1.20)	1.07(0.96-1.26)	**0.029**	1.00(0.88-1.14)	1.01(0.87-1.14)	0.915	0.99(0.87-1.13)	0.96(0.85-1.07)	**0.003**
LDL-C (mg/dl)	2.67(2.22-3.18)	2.67(2.32-3.17)	0.928	2.94(2.47-3.48)	2.98(2.42-3.50)	0.773	3.03(2.52-3.55)	3.06(2.50-3.58)	0.628
APOA (g/l)	1.02(0.92-1.17)	1.06(0.94-1.22)	0.114	1.02(0.92-1.15)	1.00(0.89-1.09)	0.106	1.04(0.93-1.16)	1.01(0.93-1.14)	0.376
APOB (g/l)	0.77(0.65-0.89)	0.74(0.65-0.88)	0.489	0.83(0.73-0.97)	0.84(0.75-0.97)	0.725	0.88(0.76-0.99)	0.87(0.76-1.01)	0.951
APOE (mg/dl)	3.78(3.17-4.71)	3.62(2.89-4.51)	0.163	4.24(3.48-5.26)	4.26(3.23-5.83)	0.916	4.47(3.61-5.70)	4.47(3.63-5.75)	0.954
FBG (mmol/l)	5.09(4.78-5.41)	5.05(4.66-5.35)	0.091	5.25(4.90-5.68)	5.19(4.84-5.50)	0.327	5.40(5.03-6.00)	5.43(5.05-5.97)	0.952

FIN (uU/ml)	7.70(5.05-11.36)	7.4(4.92-10.33)	0.522		10.74(7.47-15.25)	10.34(7.36-14.75)	0.347	13.04(8.90-19.08)	13.14(9.27-19.66)	0.493
HOMA-IR	1.74(1.11-2.64)	1.62(1.01-2.36)	0.376		2.57(1.73-3.71)	2.35(1.72-3.48)	0.331	3.12(2.09-4.89)	3.29(2.17-4.78)	0.671
**Sleep apnea**										
AHI	2.0(0.8-3.4)	1.9(0.9-3.4)	0.610		21.7(18.2-25.6)	21.7(18.2-25.7)	0.851	57.6(44.9-69.7)	58.8(46.2-73.1)	0.127
ODI	2.2(0.9-3.8)	2.2(1.0-4.1)	0.789		22.1(17.1-27.8)	23(18.3-29.0)	0.274	57.3(43.7-70.9)	59.2(43.8-73.8)	0.150
Minimum SaO2 %	93(89-95)	92(88-95)	0.122		83(78-87)	82(75-86)	**0.01**	71(63-79)	72(63-80)	0.212
MAI	13.95(9.20-22.05)	13.95(8.03-23.95)	0.682		21.1(12.9-31.4)	22.9(14.1-32.5)	0.570	35.4(19.1-54.5)	36.6(21.2-55.8)	0.232

Supplementary Table S3 Interactions of residue pairs between proteins in the two complexes

	Residue pairs	
	Wild Type<br>ANGPTL4 and ACACA	Mutant ANGTPL4 T266M<br>and ACACA
hydrogen bonding	Arg 1624 - Asp 221	Arg 1624 – Asp 221
	Lys 1648 - Gln 288	Lys 1648 - Gln 288
	Thr 1655 - Gly 313	Thr 1655 - Gly 313
	Gly 1995 - Thr 316	Gly 1995 - Thr 316
	Asp 1618 - Arg 197	Asp 1618 - Arg 380
	Asp 1924 – Asn 270	Glu 1927 - Ser 290
	Glu 1927 - Ser 290	Glu 1927 - Thr 305
	Glu 1927 - Thr 305	-
salt-bridges	Arg 1624 - Asp 221<br>[NE-OD1]	Asp 1618- Arg 197<br>[OD1-NH2]
	Arg 1624 - Asp 221<br>[NH2-OD1]	Asp 1924 - Arg 272<br>[OD1-NH1]
	Asp 1618 - Arg 197<br>[OD1-NH1]	Asp1924 - Arg 272<br>[OD2-NH1]
	Asp 1618 - Arg 197<br>[OD1-NH2]	-

Figure legends

Supplementary Figure S1. Flow chart of participant enrolment for tests of serum ANGPTL4 levels. In total, 6,433 patients from the Shanghai Sleep Health Study. Finally, 125 patients met the inclusion criteria and were included in the current study.

Supplementary Figure S2. Flow chart of participant enrolment for testing ANGPTL4 T266M. In total, 5,443 patients from the genomic database were selected. Finally, 4,455 patients met the inclusion criteria and were included in the current study.

Supplementary Figure S3. KEGG metabolic pathway for ANGPTL4.

Supplementary Figure S4. KEGG metabolic pathway for ACACA.

Supplementary Figure S5. Protein-protein interactions (PPIs) between human ANGPTL4 and ACACA. PPI diagram shows interactions between ANGPTL4 and ACACA.
