# Supplementary figures and images for: T266M variants of ANGPTL4 improve lipid metabolism by modifying their binding affinity to acetyl-CoA carboxylase in obstructive sleep apnea
Source: Ann Med. 2024 Apr 4;56(1):2337740. doi: 10.1080/07853890.2024.2337740 (PMC10997356; doi:10.1080/07853890.2024.2337740)

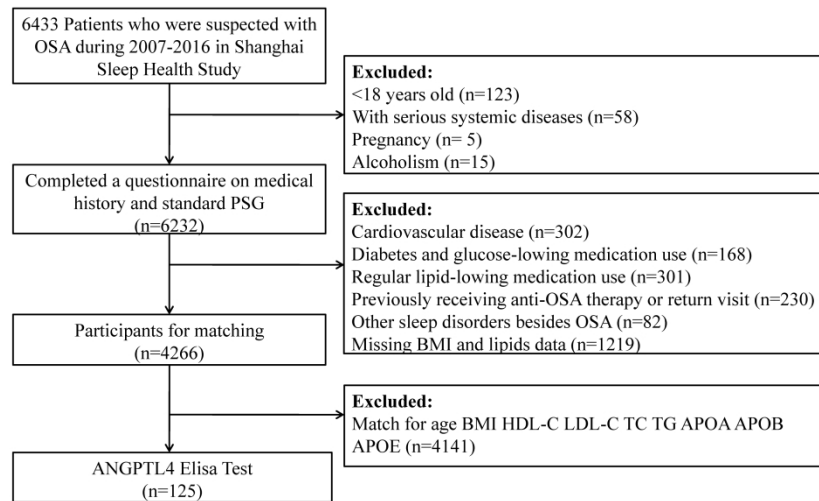

338x190mm (300 x 300 DPI)

Supplement: Supplemental Material [file IANN_A_2337740_SM3493.zip › Supplementary Figure S1.pdf]

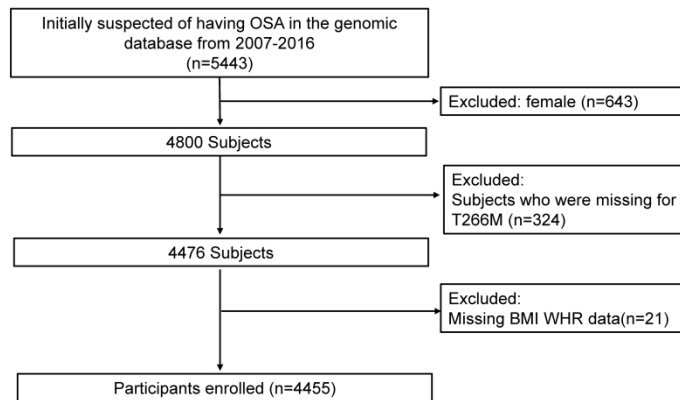

338x190mm (300 x 300 DPI)

Supplement: Supplemental Material [file IANN_A_2337740_SM3493.zip › Supplementary Figure S2.pdf]

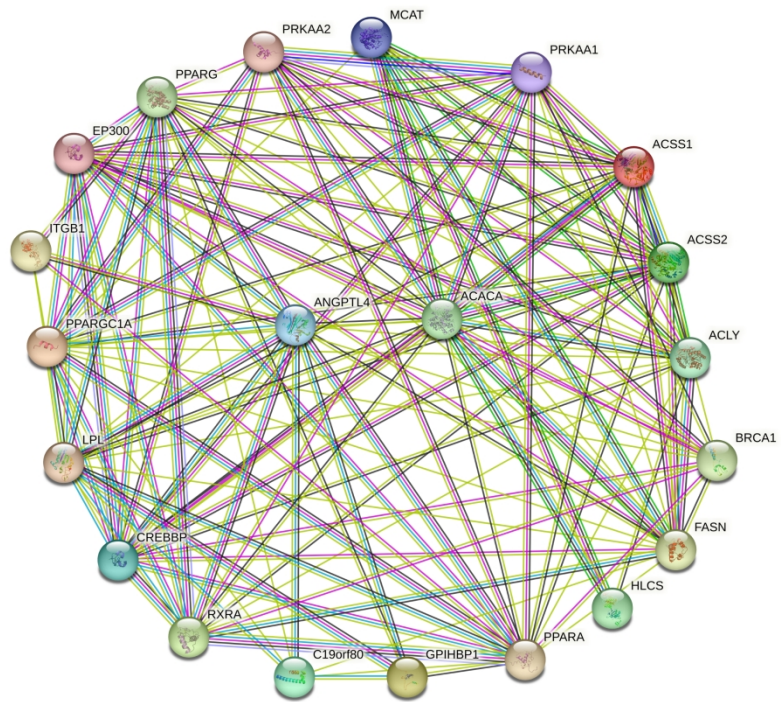

258x183mm (300 x 300 DPI)

Supplement: Supplemental Material [file IANN_A_2337740_SM3493.zip › Supplementary Figure S5.pdf]

FATTY ACID BIOSYNTHESIS

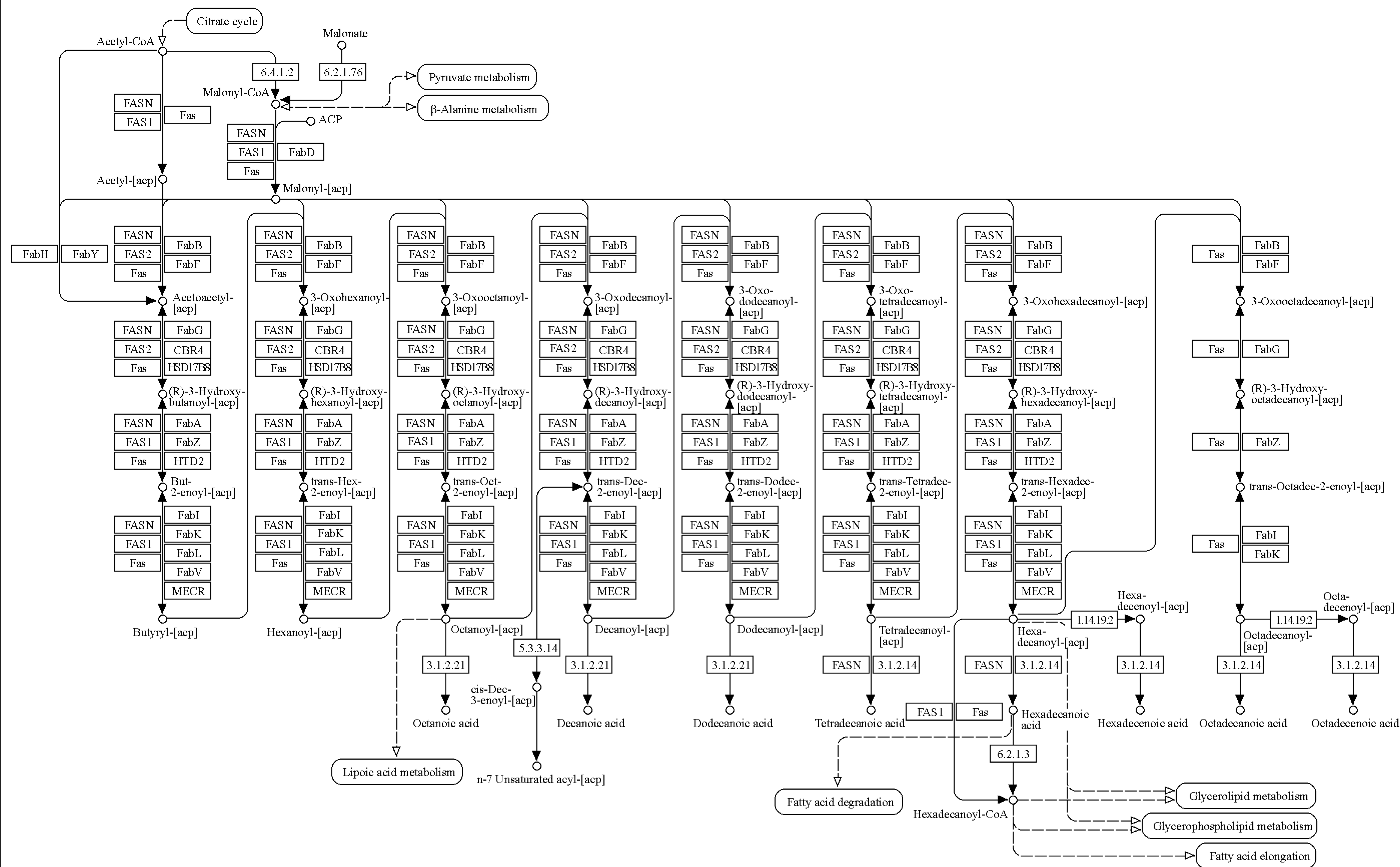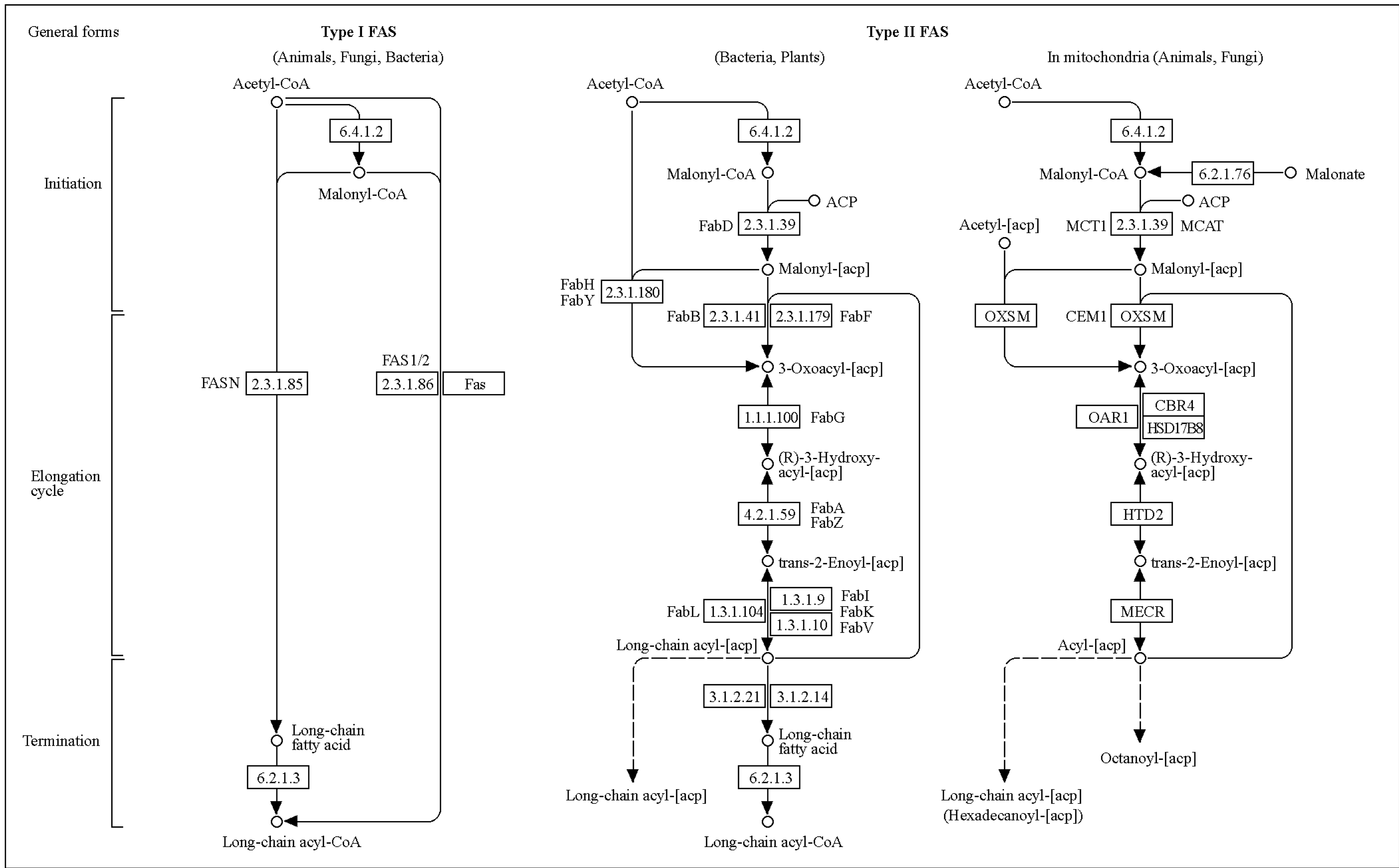

Supplement: Supplemental Material [file IANN_A_2337740_SM3493.zip › Supplementary_Figure_S4 (1).pdf]
